# Body placement of inertial measurement units differentially affects physical activity assessment accuracy in drug-naïve Parkinson’s disease

**DOI:** 10.1038/s41598-026-55099-3

**Published:** 2026-06-05

**Authors:** Corina Maetzler, Masoud Abedinifar, Vaishali Vinod, Julius Welzel, Eva Schaeffer, Rezzak Yilmaz, Markus A. Hobert, Clint Hansen, Franck Durif, Jean-Christophe Corvol, Olivier Rascol, Stefanie Behnke, Werner Poewe, Kirsten E. Zeuner, Evzen Ruzicka, Joaquim J. Ferreira, Robbin Romijnders, Daniela Berg, David Devos, Caroline Moreau, Franziska Hopfner, Walter Maetzler

**Affiliations:** 1 Department of Neurology, University Hospital Schleswig-Holstein, Campus Kiel and Kiel University, Kiel, Germany; 2 Department of Neurology, Ankara University School of Medicine, Ankara, Turkey; 3 Department of Neurology, University Hospital Schleswig-Holstein, Campus Lübeck and University of Lübeck, Lübeck, Germany; 4 Department of Neurology, CNRS, CHU Clermont-Ferrand, Institut Pascal, Université Clermont-Auvergne, 63001 Clermont-Ferrand, France; 5 Department of Neurology, CIC Neurosciences, Hôpital Pitié-Salpêtrière, Sorbonne Université, Institut du Cerveau-Paris Brain Institute-ICM, Inserm, CNRS, Assistance Publique Hôpitaux de Paris, NS-Park/FCRIN Network, Paris, France; 6 Departments of Clinical Pharmacology and Neurosciences, Clinical Investigation Center CIC1436, NS-Park/FCRIN Network, NeuroToul COEN Center, University Hospital of Toulouse, INSERM and University of Toulouse, Toulouse, France; 7 Department of Neurology, Klinik Sulzbach, Knappschaftsklinikum Saar, Sulzbach, Germany; 8 Department of Neurology, Innsbruck Medical University, Anichstrasse 35, 6020 Innsbruck, Austria; 9 Charles University and General University Hospital, Prague, Czechia; 10 Laboratory of Clinical Pharmacology and Therapeutics, Faculdade de Medicina, Universidade de Lisboa, Lisbon, Portugal; 11 CNS-Campus Neurológico, Torres Vedras, Portugal; 12 Department of Medical Pharmacology, Lille University, Lille Neurosciences and Cognition, INSERM 1172, CHU-Lille, NS-Park/FCRIN Clinical Research Network, Lille, France; 13 Neurology and Movement Disorders Department, Lille University, Lille Neurosciences and Cognition, INSERM 1172, CHU-Lille, NS-Park/FCRIN Clinical Research Network, Lille, France; 14 Department of Neurology, University Hospital of Munich, Ludwig-Maximilians-Universität (LMU) Munich, Munich, Germany

**Keywords:** Drug-naïve people with Parkinson’s disease, ENMO, IMU, MET, Early Parkinson’s disease, Health care, Medical research, Neurology, Neuroscience

## Abstract

Parkinson’s disease (PD) affects physical activity, and physical activity reduces the burden of PD. Although shown in studies using inertial measurement units (IMU), it remains unclear at which position physical activity change can best be detected in this population. Within the FAIRPARK-II trial, a subgroup of 25 newly diagnosed persons with PD (pwPD) not taking disease-specific medication yet documented their physical activity and, in parallel, wore IMUs on the most affected ankle, wrist and the lower back for two weeks. Participant-reported physical activity was transformed into Metabolic Equivalents of Tasks (METs) in 15-minute intervals using The Compendium of Physical Activities; Euclidean Norm Minus One (ENMO) values were calculated and averaged over the same intervals for the IMU data. Data of at least 3 days with at least four simultaneous 15-minute epochs of both valid IMU and diary data within the time window (9.00 to 18.00) per participant was included, resulting in a total of 8,494 15-minute epochs used for this analysis. Root mean square error (RMSE) values were calculated between scaled normalized IMU-derived ENMO and normalized MET values for each of the nine IMU-MET combinations (three IMU positions × three MET intensity levels). The wrist and lower back IMU showed comparable RMSE values across all MET intensity levels, with both IMU positions showing lower RMSE values than the ankle position. Tremor affected RMSE negatively, whereby the lower back position may be slightly favorable for the assessment of physical activity in those with tremor. This prospective longitudinal dataset from a very rare cohort provides novel insights into the assessment of physical activity during the earliest clinically evident phase of Parkinson’s disease without disease-specific medication, which may inform future clinical trials and observational studies.

**Supplementary Information:**

The online version contains supplementary material available at 10.1038/s41598-026-55099-3.

## Introduction

 Physical activity is any bodily movement produced by skeletal muscles that results in energy expenditure^1^. On the one hand, reduced physical activity is associated with major non-communicable diseases like coronary heart disease, type 2 diabetes, and some cancers, and is the fourth leading risk factor for global mortality (6% of deaths globally^2^. In Parkinson’s disease (PD), symptoms such as bradykinesia, rigidity, postural instability, fatigue, depression and apathy often result in reduced physical activity. This has been shown for early^3–5^, moderate^6,7^ and advanced stages^8^ and, for prediagnostic phases of the disease^9^. On the other hand, an increase in physical activity reduces symptom burden of persons with PD (pwPD)^10^, improves quality of life^11^ and has a positive influence on disease course^12^. Overall, this makes physical activity an important and clinically highly relevant predictive and outcome parameter also in pwPD^13^.

During previous decades, physical activity was mainly assessed using self-administered questionnaires such as the International Physical Activity Questionnaire (IPAQ)^14^ and the Physical Activity Scale in the Elderly (PASE)^14,15^. From these instruments, energy expenditure has usually been calculated with metabolic equivalents of tasks (METs), which reflects the energy cost of a physical activity as a multiple of the resting metabolic rate^16^. In 1993, the Compendium of Physical Activities was developed, a comprehensive list of physical activities encoded in a standardized system^16^ that can be used for the granular individual collection and evaluation of METs. Although it does not directly measure energy expenditure, as would be expected from a gold-standard measure, its results can be used as reference values when developing new assessment tools.

As self-administered questionnaires have downsides, such as subjectivity, recall bias and relatively large effort to collect data, novel methods have been explored. One of them is the assessment with mobile digital devices, with inertial measurement units (IMUs) being the most used technology that can be found, e.g., in basically all smartphones and smartwatches currently available^17^. IMUs usually measure acceleration, angular velocity and magnetic orientation. For activity assessment, data is often broken down into “activity counts” per defined period of time (epoch) which represent an estimate of the intensity of the activities conducted during these epochs. However, Euclidean Norm Minus One (ENMO) has also been defined as the metrics of choice^18–22^. ENMO values quantify physical movement from acceleration signals by isolating the dynamic component while excluding the effect of gravity^23–25^.

In research, IMUs have been positioned on different body positions to assess physical activity in everyday life environment, over longer periods, in the frame of days and weeks. White et al.^26^ and Gilby et al.^27^ report about physical activity assessment using IMUs on the wrist in healthy adults and people with newly diagnosed PD, respectively. Van Schooten et al.^28^, Soangra et al.^29^ and van Uem et al.^30^ used IMUs on the lower back for long-term assessment of physical activity in the home environment in older adults, healthy younger and healthy older adults, and pwPD, respectively. Some studies have used IMUs on the ankle to assess physical activity in healthy youth^31^ and in healthy adults^32^, however group sizes were small and simulated daily activities were performed in a lab environment. To our best knowledge, there are currently only few studies available that evaluated the performance of different positions of IMUs for this purpose. Duncan et al.^33^ let older adults perform five-minute bouts of activity: lay supine, seated reading, slow walking, medium walking, fast walking, folding laundry, sweeping and stationary cycling, and found ankle and waist positions most favorable. Another study^34^ examined older adults with various neurological conditions performing activities of daily living (single and dual-task walking, morning routines, household tasks and leisure activities) for 30 min. The study found that a waist-worn IMU detected the highest percentage of steps performed (as recorded on video), followed by an IMU worn on the lower back. Davoudi et al.^35^ found an IMU positioned on the hip or thigh providing the best MET estimation and activity level recognition in older adults compared to IMUs positioned on the ankle or wrist. However, no long-time assessments in real life scenarios are currently available, neither in healthy people nor in people with mobility-limiting diseases.

Therefore, we set out to compare IMU-derived data from the ankle, wrist and lower back, respectively, with reference values obtained from a detailed in-house developed physical activity diary (Supplementary Table 3) filled in over a 2-week period by pwPD who were drug-naïve to any disease-specific medication.

## Results

A total of 336 days with at least one 15-minute diary entry and 344 days with at least nine hours of recorded sensor data were provided by the 25 pwPD included in this study. These days started at 09:00 or earlier and ended at 18:00 or later. Of these days, 78 days of diary data and 86 days of recorded IMU data had to be excluded, as these did not contain at least four 15-minute epochs of IMU and diary data simultaneously. Therefore, 258 days fulfilled our inclusion criterion, and 8,494 15-minute epochs could be included in the analysis (with a minimum of 113 and a maximum of 518 epochs per participant). All participants showed a lateralisation of PD-associated motor symptoms.

The correction factors resulting from the Nelder-Mead optimisation ranged from 0.633 to 1.084 across all IMU-MET combinations (Supplementary Table 1). Wrist ENMO amplitudes were systematically higher relative to normalized MET and thus required stronger downward corrections (0.633–0.724), whereas ankle and lower back sensors required smaller corrections (0.929–1.084).

**Fig. 1:**
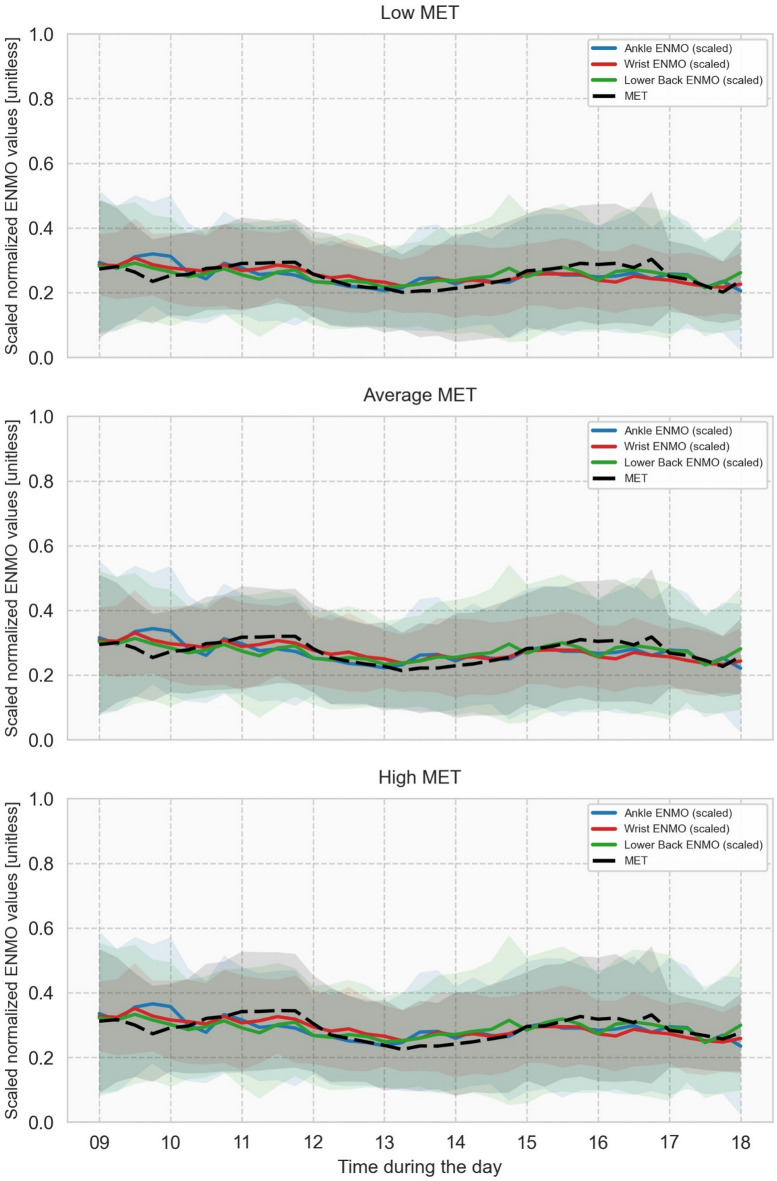
Comparison of sensor-derived physical activity values with reference values throughout the day. Group-mean temporal profiles of scaled normalized ENMO and corresponding normalized MET intensity levels across the daytime window (09:00 to 18:00) for 25 people with Parkinson’s disease (pwPD), following Nelder-Mead scaling optimization. Each subplot shows one MET intensity level: low MET, average MET, and high MET. Solid lines represent scaled ENMO from three inertial measurement unit (IMU) positions: Ankle (blue), Wrist (red), and Lower Back (green). Dashed lines represent the normalized MET reference for each intensity level. Shaded areas indicate ± 1 SD across participants. Scaling factors were derived by minimizing RMSE between group-mean signals using the Nelder-Mead algorithm, with the model MET = α × ENMO.

The normalized MET values (from now on called “MET values”) and the scaled normalized ENMO values (from now on called “ENMO values”) for each IMU position (ankle, wrist and lower back) across the daytime period are presented in Fig. 1. For each of the three MET levels (low, average, and high MET), the figure shows the group-mean temporal profiles together with ± 1 SD across the 25 pwPD for each 15-minute time window from 09:00 to 18:00, illustrating both the overall temporal trend and the interindividual variability in activity patterns throughout the day.

**Fig. 2:**
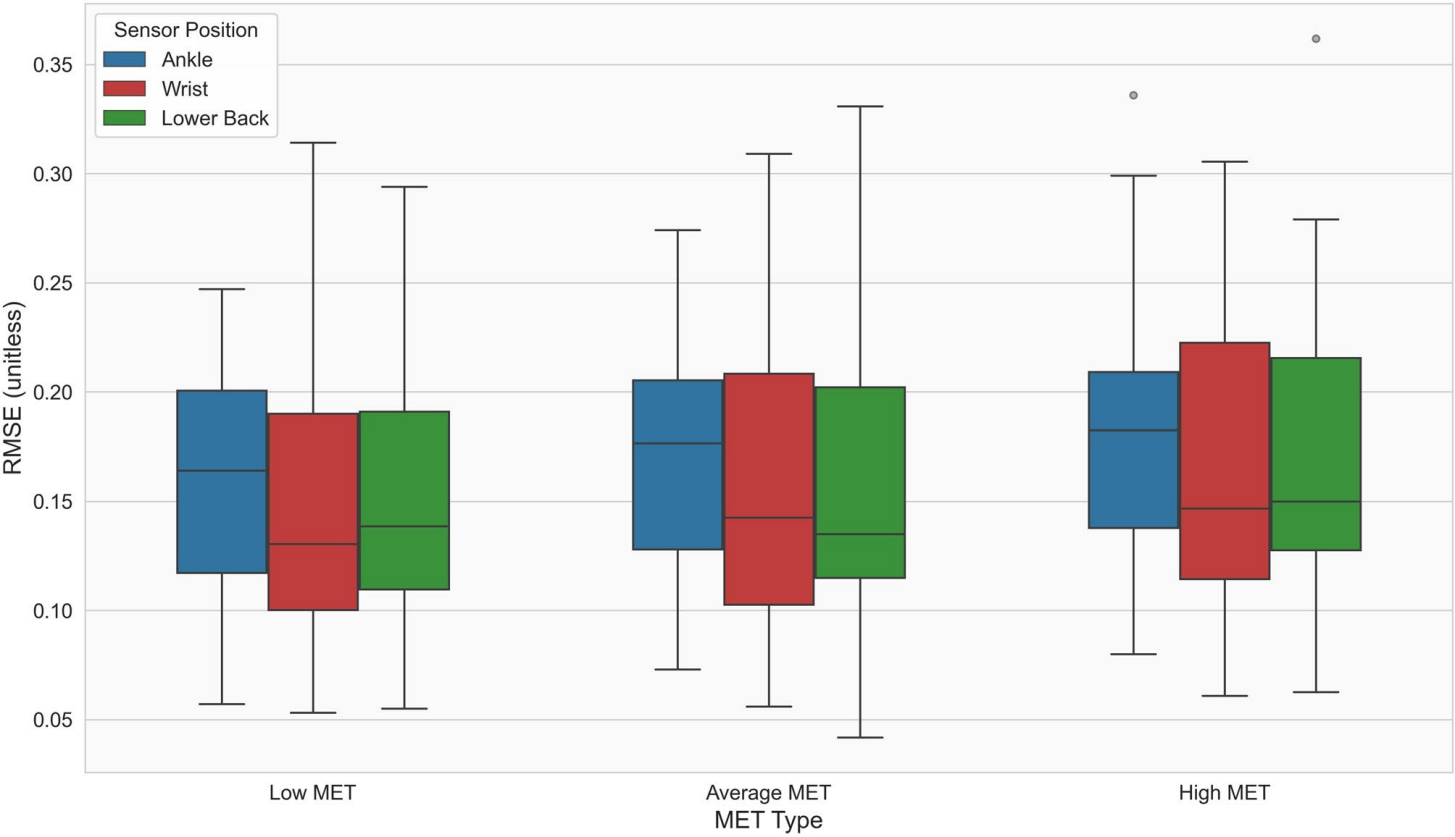
Comparison of sensor-derived physical activity values with reference values. Distribution of root mean squared errors (RMSE) between scaled normalized ENMO and normalized MET values across 25 people with Parkinson`s disease (pwPD), stratified by MET intensity level (low, average, high MET) and inertial measurement unit (IMU) position, following application of the Nelder-Mead optimal correction factors. Box plots show the median and interquartile range (IQR); individual outliers are shown as grey circles. IMU positions are color-coded: Ankle (blue), Wrist (red), Lower Back (green).

Figure 2 and Table 1 present the RMSE values for the different IMU positions across all three MET levels. Across all MET levels, the lowest and most comparable RMSE values were yielded by the wrist and lower back IMU positions. The ankle position showed RMSE values that were 20–25 per cent higher across all three MET levels.

**Table 1:** Root mean squared errors of inertial measurement unit-derived values, compared to diary values.

MET type	Sensor	Median	Min	Max
Low MET	Ankle	0.164	0.057	0.247
	Wrist	0.130	0.053	0.314
	Lower back	0.138	0.055	0.294
Average MET	Ankle	0.176	0.073	0.274
	Wrist	0.142	0.056	0.309
	Lower back	0.135	0.041	0.330
High MET	Ankle	0.182	0.080	0.335
	Wrist	0.146	0.061	0.306
	Lower back	0.150	0.062	0.361

We also analysed the results based on whether or not tremor was present, at an exploratory level. Five participants did not exhibit tremor; 20 participants exhibited tremor in their most affected upper extremity. After splitting the group into subgroups with no/minimal tremor (*n* = 14) and stronger tremor (*n* = 11), RMSE values at low, average, and high MET levels were extracted for the wrist and lower back IMU positions (but not for the ankle position, as this position was inferior in the overall analysis and only one person had leg tremor). Visual inspection did not show a systematic increase of the ENMO curves over the MET curves in the stronger tremor subgroup compared to the no/minimal tremor subgroup (Supplementary Fig. 1). Quantitative analysis with medians and ranges revealed that RMSE values were generally around 60 per cent higher in the stronger tremor subgroup than in the no/minimal tremor subgroup for all MET levels and IMU positions. The wrist IMU position performed slightly better than the lower back position in the no/minimal tremor subgroup, and the reverse was true for the stronger tremor subgroup. Results are presented in Supplementary Table 2.

## Discussion

In our study, IMUs on three different positions of the body were all able to assess physical activity in drug-naïve, early pwPD relatively well. This result confirms findings from previous studies in older adults^35^ and persons in various stages of PD^36,37^. The reviews by Correno et al.^36^ and Sica et al.^37^ showed that, in both clinical and daily life environments, the physical activity of pwPD at different stages of the disease was best recorded by IMUs close to the centre of mass (i.e. on the waist or lower back). Davoudi et al.^35^ showed that, in a population of older adults, the IMU placed on the hip was the best position for MET estimation and activity level recognition.

We assessed, as the reference value, physical activity with a diary on a novel level of granularity and over a long evaluation period and used, in parallel, IMUs on three different body positions to enable a comparison between the subjective and objective evaluation of physical activity. We recorded physical activity using subjective and objective measurement methods over a relatively long period of time. Using a detailed activity diary, individual recording of everyday movement patterns was possible. This is an improvement compared to most of the currently used diaries, also because the information was collected a couple of times during a day (which comes close to an ecological momentary assessment, EMA)^38,39^, efficiently reflecting the activity that was done only a few hours or minutes before it was documented. Furthermore, this study collected data from three different body positions, all of which have high potential for accurately assessing physical activity.

We also used a sophisticated evaluation protocol that allows a direct application of correction factors between the two methods, and the evaluation of the direction of error the IMU position has compared to the reference value. This study provides also correction factors for three body positions and a clearly defined PD subgroup (drug-naïve, close after diagnosis). Concerning the specific RMSE values of the IMUs, an advantage of our approach is that these values allow a simple interpretation of under- and overestimation of physical activity, as presented by the reference value (the diary), on a particularly granular level of 15-minute epochs.

How could the results be interpreted? It seems that positioning IMUs on the wrist and lower back is equally suitable for the monitoring of physical activity in the very early clinical phase of PD, providing similar results: The RMSE values for these positions were consistently below 15 per cent. This result is promising. However, the equal “performance” of wrist and lower back position was somewhat surprising. Previous studies in older adults^35^ and pwPD^40^ have shown that when using a single IMU to quantify physical activity, an IMU positioned close to the center of mass provides the most valid and reliable results in a laboratory^35^ and in a free-living-like laboratory setting^40^. This also makes sense from a pathomechanistic point of view: Accelerations measured at this body position best represent daily activities, even in a sedentary state^41,42^. However, compared to IMUs on the lower back, wrist-worn IMUs have been shown to measure hand and upper-limb activity more precisely, as shown in people with dementia^43^.

The relatively high RMSE for ankle position was mainly due to the overestimation of physical activity at the start of the day, between approximately 09:30 and 10:30 (see Fig. 1). One possible explanation for this is that this position reflects activities such as using the restroom and kitchen, which involve relatively high levels of shuffling and therefore can lead to a higher proportion of ankle movements compared to overall body movement.

Breaking down the data into low, average and high MET levels did not provide any additional relevant results. This level of granularity was introduced because some study participants had recorded performing activities that require varying amounts of energy simultaneously, for example housekeeping and watching TV. The comparable RMSE values of IMU positions across all MET levels suggest that the analytical approach presented here, which includes group-level scaling and normalization and the use of correction factors (see Supplementary Table 1), allows for an adequate measurement of physical activity, regardless of which MET level is used as the reference value. This is particularly true when the IMUs are positioned on the wrist or lower back.

The presence of tremor significantly impacted the accuracy with which the IMU detected physical activity as evaluated with the reference measure. RMSE values from participants exhibiting stronger tremors were substantially higher than those from participants exhibiting minimal or no tremors. One possible explanation is that tremor itself may lead to higher ENMO values (as one would expect), but it may also lead to reduced movement; for example, people may put their trembling hand in their pocket when walking or standing to hide the symptom. Although, to our knowledge, no literature specifically addresses the association between tremor, compensatory movement strategies and concealment behaviours, there is clear evidence that tremor leads to embarrassment, stigma, emotional distress and social interaction difficulties, interfering with daily activities and professional life^44^. This is particularly evident when the symptom first appears^45^. It is therefore tempting to speculate that the substantially increased RMSE, without a relevant parallel increase in ENMO values, in the subgroup with stronger tremor reflects a complex human symptom in combination with compensatory and adaptive strategies. Further studies are needed to investigate this phenomenon in more depth. It is also interesting to note that the wrist position is likely to be more advantageous for evaluating physical activity in the absence of or with minimal tremor, whereas the lower back position is preferable when tremor is present. The reason is probably that tremor has a greater influence on movements of the extremities than on axial movement^46^.

This is, to our best knowledge, the first study investigating a group of drug-naïve pwPD with IMUs at different body positions over a long period of time. This dataset enables daily activity to be analysed at a new level of detail and allows the data from the different positions to be combined (e.g. for kinematic analyses^47^. Furthermore, this dataset could be used in future to analyse specific movements, such as turns and transitions, and to distinguish between controls and PD in everyday situations. Ultimately, as this is a prospective longitudinal dataset with a rare cohort, it will enable the analysis of changes in physical activity and adaptation strategies during the earliest and “native” clinically evident phase of the disease, over a long period, without any treatment-related components, such as fluctuations and dyskinesias.

## Methods

### Participants and study protocol

In total, 25 newly diagnosed pwPD without disease-specific medication (9 female, 36%) took part in this study. Table 2 provides demographic and clinical parameters. Ethics committees in each country approved the conduct of the trial. All the participants provided written informed consent before screening.

**Table 2:** Demographic and clinical parameters.

	Mean	Std. deviation	Minimum	Maximum
Age [years]	61	10	41	74
Disease duration [months]	6	3	1	11
Hoehn &Yahr stage (1–5)	1.7	0.6	1	2
MDS-UPDRS III (0–132)	18	5	9	29
Tremor subitems (0–12)	1.6	1.2	0	5

MDS-UPDRS III: Motor part of the Movement Disorders Society-Unified Parkinson’s Diseases Rating Scale. The tremor subgroups were formed based on the sum scores of items 3.15, 3.16 and 3.17 of the MDS-UPDRS Part III, as determined by the participants’ most affected side.

PwPD were recruited in the frame of the FAIRPARK-II study, a multicentric phase-2 randomized, placebo-controlled trial that evaluated the efficacy and safety of deferiprone^48^. The participating sites of the sub-study were Lille, Toulouse, Paris, Clermont-Ferrand (all in France), Kiel, Homburg (Germany), Innsbruck (Austria), Prague (Czech Republic), and Lisbon (Portugal). FAIRPARK-II was coordinated by Prof. Devos (sponsor) with the strategic support of the “NS-PARK” network belonging to the National Clinical Research Infrastructure “F-CRIN” and the European Clinical Research Organization “ECRIN” and conducted in accordance with the International Council for Harmonisation Good Clinical Practice guidelines and the ethical principles of the Declaration of Helsinki. Interaction with competent authorities and an independent ethics committee was performed by the Sponsor for France and by the national ECRIN partners for other EU countries. The sponsor’s ethics application was approved by the Comité de Protection des Personnes NORD OUEST IV under the reference number CPP 15/53. In the trial, 3 visits were performed, which were also the time points when the sub-study collected data over a maximum of 2 weeks each: weeks 0–1 (T1, the data collection started the same day as deferiprone intake), 35–36 (T2, at the end of deferiprone intake) and 39–40 (T3, after the deferiprone washing out phase). This study presents data from T1 of participants from the verum (12) and of the placebo group (13).

### Physical activity assessment with diaries and extraction of energy expenditure values

The diary used in this study is based on the Zutphen Physical Activity Questionnaire (ZPAQ)^49^, the Physical Activity Scale for individuals with Physical Disabilities (PASIPD)^50^, the Short Questionnaire to assess health-enhancing physical activity (SQUASH)^51^ and the Physical Activity Scale for the Elderly (PASE)^15^. We used a similar structure assessing type and intensity of the respective activity. However, an adaptation was necessary as all the above-mentioned instruments collect information about the previous week up to the previous months and were not designed to collect information in a “life” setting. For the study, participants were asked to record the type, start time, end time and intensity (mild, moderate or vigorous) of their recent activities as frequently as possible throughout the day — ideally at least three times. The self-reported perception of intensity was used to assign MET values according to the activity levels provided for single activities by the Compendium. The diary was structured into the following activity sections: activities inside the house; activities around the house (e.g. gardening, tinkering, doing crafts); activities in other environments (e.g. shopping, going for a walk, playing sports, visiting friends/family, riding a bike); public transport (e.g. car driver, car passenger, motorbike/scooter rider, train passenger); sleeping and resting phases; eating phases; and IMU non-wear periods. Participants could also leave comments for each reported activity. The full version of the diary is available as Supplementary Table 3.

Diary entries were extracted and collected in a spreadsheet in time periods of at least 15 min. Each diary entry comprised the activity type, start time, end time, and self-reported intensity. Activity types were matched to the most appropriate code in the Compendium of Physical Activities (Ainsworth et al.^16^.) by two researchers (C.M. and M.A.) independently, with discrepancies resolved by consensus. The assigned Compendium MET value was then mapped to the corresponding 15-minute epoch within the 09:00–18:00 window.

### Physical activity assessment with Inertial Measurement Units (IMUs) and extraction of energy expenditure values

Participants wore three IMUs (RehaGait^®^, HASOMED, Magdeburg, Germany), synchronised through the *SensePark* Software (Version 1.4.9 beta, provided by HASOMED) via date-stamps, with stretch belts on the ankle, the wrist, and the lower back, respectively (please note that HASOMED no longer distributes the RehaGait^®^ devices or the SensePark software, https://hasomedscience.de/en/legacy-produkte). Ankle and wrist IMUs were positioned on the most affected side (all participants showed lateralised parkinsonism). The dimensions of the IMUs are 60 × 35 × 15 mm and each device comprises a 3-axis accelerometer (± 16 g), a 3-axis gyroscope (± 2000°/s) and a 3-axis compass (± 1.3 Gs)^52^. Data was collected at a sampling frequency of 100 Hz. Participants were asked to wear the IMUs from when they got up until they went for sleep, and devices were recharged during night. The .gdb files created by the *SensePark* software for each measurement period were first collected locally and then transferred to the local cloud storage server at Kiel University.

Acceleration data of all three axes (x, y, z) were then extracted and processed to calculate ENMO values. The raw accelerometer data were recorded in gravitational units [g]; therefore, the gravitational constant (1 g) was subtracted from the Euclidean norm to remove the static component due to gravity^25,53^, and a fourth-order low-pass Butterworth filter with a cutoff frequency of 20 Hz was applied^24,26^. Negative ENMO values were truncated to zero^25,26,53,54^. Then, the continuous ENMO signal was segmented into consecutive, non-overlapping 5-second epochs, within which the mean ENMO value was calculated^25,54^. All 5-second ENMO values were then aggregated into 15-minute epochs, with each 15-minute epoch representing the arithmetic mean of all corresponding 5-second ENMO values within that time window.

#### Data analysis

From the dataset, all participants with at least 3 days of IMU assessment (09:00 to 18:00) and with at least four simultaneously assessed 15 min epochs of diary entry per day were included in the analysis. If participants recorded parallel activities (e.g. housekeeping and watching TV), the corresponding low, average, and high MET values were derived separately for each activity and processed as three distinct MET levels.

Our objective was to evaluate the temporal misalignment between the 15-minute ENMO epochs (based on time stamp information; e.g., 9:00–9:14; 9:15 − 9:29) and the corresponding 15-minute MET epochs (based on information available from the diary) throughout the observation period, regardless of scaling issues. To this end, we took the following steps.

1. First, to enable direct comparison between ENMO and MET signals, which differ in physical units and magnitude, all data were normalized to the range [0, 1] using Min-Max scaling (MinMaxScaler, Scikit-learn library, Python)^55^, where the value ‘0’ corresponds to the observed global minimum and ‘1’ to the observed global maximum. For ENMO, normalization was performed separately for each sensor position (ankle, wrist, and lower back) using sensor-specific global minimum and maximum values calculated across all subjects and all daytime time slots (09:00 to 18:00). For MET, the three intensity levels (low, average, and high MET) were normalized jointly using a single shared Min-Max transformation.
2. Second, to correct for residual amplitude mismatch between the normalized ENMO and normalized MET signals, which may persist after normalization because the two signals capture fundamentally different physical quantities, a linear correction factor α was introduced following the model MET = α × ENMO. For each of the nine sensor-MET combinations (three sensor positions × three MET levels), α was determined by minimizing the RMSE between the group-mean normalized ENMO and the corresponding group-mean normalized MET signal using the Nelder-Mead simplex optimization algorithm^56^(SciPy opt.minimize). Optimization was initialized with a correction factor equal to the ratio of the two group-mean signal averages to ensure convergence near the true optimum, and default convergence tolerances (xatol and fatol) were applied.
3. Third, to quantify individual-level ENMO-MET agreement, RMSE values were computed for each pwPD using the α-corrected normalized ENMO signal, against the corresponding normalized MET signal. This yielded a distribution of 25 RMSE values per ENMO-MET combination (Fig. 2). These RMSE values were used for all analyses presented in the main document and in the supplementary material.
4. To analyse the influence of tremor on the main results, the 25 datasets were split into two subgroups: One with no or minimal tremor on the most affected side (defined by the total tremor score of items 3.15, 3.16 and 3.17 of MDS-UPDRS Part III ≤ 1, *N* = 14) and one with stronger tremor on the most affected side (score > 1, *N* = 11).

### Limitations

One of the limitations of this study is that only one IMU from a single company was used, and future studies may use various devices to address this issue^57^. Another limitation is that we did not have complete diary records for the entire period when IMU data was collected, which could introduce bias. We also asked the participants to record their activities in the diary ‘at least three times per day’, which may result in varying levels of detail and accuracy in the diary data across participants. It should also be noted that MET estimates based on diary entries are inherently subjective. Furthermore, it would have been interesting to compare data from ankle and wrist IMUs worn on the most-affected dominant, and the most-affected non-dominant side, respectively. We suggest to further investigate in this topic in future studies with a larger sample and in other PD subpopulations, as well as in further conditions where physical activity is affected. Future studies may also investigate our hypotheses about how and why tremor influences RMSE values.

## Conclusion

This study on a drug-naïve PD cohort who had recently been diagnosed compared physical activity values obtained from IMUs worn at the ankle, wrist and lower back against data from a detailed physical activity diary. All three sensor positions reflected the general diurnal activity profile captured by self-reports but differed in their agreement with diary-derived MET values. Our findings underline the importance of sensor placement when assessing daily physical activity in pwPD and suggest that wrist and lower back placement may offer the best compromise between accuracy and robustness in real-world monitoring, with slight advantages for the wrist position in those without tremor, and slight advantages for the lower back position in those with tremor. Future studies should confirm these findings in larger and more advanced cohorts, different activity contexts and by using more granular assessment methodology.

## Supplementary Information

Below is the link to the electronic supplementary material.

Supplementary Material 1

Supplementary Material 2

## Data Availability

The data for this study is available from the corresponding author upon reasonable request.
