# Supplementary material for: Body placement of inertial measurement units differentially affects physical activity assessment accuracy in drug-naïve Parkinson’s disease
Source: Sci Rep. 2026 Jun 5;16:17511. doi: 10.1038/s41598-026-55099-3 (PMC13241497; doi:10.1038/s41598-026-55099-3)
Supplement: Supplementary file 1 — Supplementary Material 1 [file 41598_2026_55099_MOESM1_ESM.docx]

**FAIRPARK-II study group** (with the support of the Lille University Hospital and NSPark/FCRIN clinical research network, ECRIN, www.fairpark2.eu)

**Centres and list of investigators:**

**23 centers**

| **Country** | **Sites** | **Principal Investigator** |
| --- | --- | --- |
| France | Lille | Pr Defebvre Luc |
|  | Toulouse | Pr Rascol Olivier |
|  | Strasbourg | Pr Tranchant Christine |
|  | Marseille | Dr Eusebio Alexandre |
|  | Lyon | Pr Thobois Stéphane |
|  | APHP (Paris) | Pr Corvol Jean-Christophe |
|  | Clermont-Ferrand | Pr Durif Franck |
|  | Bordeaux | Pr Meissner Wassilos |
| Spain | Hospital Clinic in Barcelona | Dr Yaroslau Compta |
|  | Germans Trias I Pujol Hospital | Dr Dolores Vilas |
|  | Hospital Sant Pau | Dr Jaime Kulisevsky |
| Austria | Medizinische Universitat Innsbruck | Pr Werner Poewe |
| Czech Republic | Univerzita Karlova V Praze | Pr Evzen Ruzicka |
| Portugal | Hospital da Senhora da Oliveira, Guimarães | Dr Miguel Gago |
|  | Centro Hospitalar e Universitario de Coimbra | Dr Cristina Januario |
|  | Centro Hospitalar Lisboa Norte | Pr Miguel Vilhena Soares Coelho |
| Germany | Christian-Albrechts-Universität zu Kiel | Pr Daniela Berg |
|  | Homburg | Pr Stefanie Behnke |
|  | Universität Rostock | Pr Uwe Walter |
| UK | Cambridge | Dr Paul Worth |
|  | Newcastle | Pr Nicola Pavese |
| Netherlands | Radboudumc | Dr Bart Post |
|  | Academic Central Center | Pr Rob de Bie |

**The investigators of the 23 centres:**

Abbruzzese Giovanni

Accart Bertrand

Allain Marie-Anne

Anheim Mathieu

Ardigo Diego

Aracil-Bolaños Ignacio

Baba Paul

Bakker Martijn

Balzer-Geldsetzer Monika

Bargalló Núria

Barone Paolo

Basenau Sandra

Beliveau Vincent

Benchetrit Eve

Berg Daniela

Best Laura

Bloem Bas

Bonicel Robin

Boraud

Bordet Regis

Bouca Raquel

Bourdain Frédéric

Bouzas Jimena

Brefel-Courbon Christine

Bubenheim Michael

Burn David,

Bush Ashley I.

Cabantchik Ioav

Calvas Fabienne

Cámara Ana

Campolongo Antonia

Carrière Nicolas

Chaigneau Véronique

Collin Matthieu

Compta Yaroslau

Connelly John

Cormier-Dequaire Florence

Corvol Jean-Christophe

Cranston Amy

Dean Rory

De Bie Rob M.A.

De Marzi Roberto

Defebvre Luc

Degos Bertrand

Demotes Jacques

Dellapina Estelle

Deplanque Dominique

Devedjian Jean-Christophe

Devos David

Dexter David

Dodel Richard

Dongmo Carole

Duce James

Duhamel Alain

Dupouy Julia

Durif Franck

Dusek Petr

El Mountassir Fouzia

Eusebio Alexandre

Eyvrard Frédéric

Fernández Manel

Ferreira Joaquim

Forni Gian Luca

Foster Victoria

Foubert-Samier Alexandra

Fradette Caroline

Fréville Laëtitia

Galitzky Monique

Gaudebout Cecile

Gelé Patrick

Giladi Nir

Grabli David

Gleixner Franck

Grolez Guillaume

Guyon Pauline

Habert Marie-Odile

Harroch Estelle

Hartmann Andreas

Hirsch Denise

Hisbergues Michael

Hobert Markus A.

Hopfner Franziska

Jurado Camille

Kaiser Andreas

Keen Gill

Kouassi Nadège

Labreuch Julien

Lacomblez Lucette

Lagha Boukbiza Ouhaid

Lanthaler Barbara

Lechatellier Gilles

Le Forestier Nadine

Lehmann Fred

Lloret Teresa

Le Naour

Le Toullec Benjamin

Locatelli Maxime

Löhle Matthias

Lomeña

Longato Nadine

Lützen Ulf

McNichol Ann

Maetzler Corina

Maetzler Walter

Mahlknecht Philipp

Mangone Graziella

Marín-Lahoz Juan

Mariani Louise-Laure

Marques Ana

Matei Mihaela

Matthias Löhle

Maucourt Bacchi Emilie

Meissner Wassilios

Michon Amelie

Moreau Caroline

Nardocci Nardo

Nosal Florence

Nyholm Dag

Oeckl Patrick

Oravska Irena

Ory Fabienne

Otto Markus

Ouk Thavarak

Pagonabarraga Javier

Pascual-Sedano Berta

Pavese Nicola

Peball Marina

Phillips Clélie

Pineau Fanny

Planellas Lluís

Poewe Werner

Pop-Ilieva chiesi

Post Bart

Rabier Aurélie

D Olivier

Riedel Christian

Rodrigues Maura

Roullet-Solignac Isabelle

Rose Christian

Rozova Anna

Růžička Evžen

Salis Alexandra

Schäffer Eva

Scherfler Christoph

Schiefermeier Natalia

Seppi Klaus

Smagghe Delphine

Silva Tânia

Silva Pedro

Socha Julie

Souyris Corinne

Spampinato Umberto

Spino Michael

Steel Alison

Sweta Bajaj

Thalamas Claire

Teodor Danaila

Teresa Anna

Thobois Stéphane

Tison François

Tolosa Eduardo

Tranchant Christine

Tricta Fernando

Trifirò Gianluca Trifirò

Vidailhet Marie

Walter Uwe

Wang Yi

Werkmann Mario

Yilmas Rezzak

You Hana

Zeuner Kirsten
