# Supplementary material for: Body placement of inertial measurement units differentially affects physical activity assessment accuracy in drug-naïve Parkinson’s disease
Source: Sci Rep. 2026 Jun 5;16:17511. doi: 10.1038/s41598-026-55099-3 (PMC13241497; doi:10.1038/s41598-026-55099-3)
Supplement: Supplementary file 2 — Supplementary Material 2 [file 41598_2026_55099_MOESM2_ESM.docx]

Supplementary material

**Supplementary Table 1.** Correction factors for inertial measurement unit (IMU)-derived values

| **MET type** | **Ankle** | **Wrist** | **Lower back** |
| --- | --- | --- | --- |
| **Low MET** | 0.929 | 0.633 | 0.949 |
| **Average MET** | 0.998 | 0.681 | 1.019 |
| **High MET** | 1.063 | 0.724 | 1.084 |

*Correction factors (α) obtained via Nelder-Mead optimization for each Metabolic Equivalent of Tasks (MET) intensity level, separated by inertial measurement unit (IMU) position.*

**Supplementary Table 2.** Root mean squared errors (RMSE) of inertial measurement unit (IMU)-derived values compared to diary Metabolic Equivalent of Tasks (MET) values, stratified by tremor subgroup, MET type, and IMU position.

|  |  | **No/minimal tremor subgroup (N=14)** | | **Stronger tremor subgroup (N=11)** | |
| --- | --- | --- | --- | --- | --- |
| MET Type | **IMU position** | **Median** | **Min-Max** | **Median** | **Min-Max** |
| Low MET | Wrist | 0.113 | 0.060-0.237 | 0.189 | 0.053-0.314 |
|  | Lower Back | 0.129 | 0.055-0.203 | 0.189 | 0.104-0.294 |
| Average MET | Wrist | 0.113 | 0.059-0.224 | 0.203 | 0.056-0.309 |
|  | Lower Back | 0.134 | 0.042-0.210 | 0.195 | 0.098-0.331 |
| High MET | Wrist | 0.118 | 0.061-0.280 | 0.212 | 0.061-0.306 |
|  | Lower Back | 0.139 | 0.063-0.275 | 0.203 | 0.093-0.362 |

*Inertial measurement unit (IMU); Metabolic Equivalent of Tasks (MET).*

**Supplementary Table 3. Activity Diary**

# Dear participant,

Thank you for participating in this study.

You will be monitoring movements in your home environment. In order to have a useful interpretation of your collected data, we need your help, and we kindly ask you to fill out the following diary as frequently and detailed as possible.

The diary contains the following elements

| **Element** | **When to fill out?** | **Illustration** |
| --- | --- | --- |
| **Medical therapy** | At the start of each assessment period | Please fill out your (medical or non-medical) therapy (dosage, time frame).  Please also document if your medication intake deviated from the regular medical therapy. |
| **Activities** | Daily | Please document any physical (walking, biking, eating, housekeeping) or non-physical (reading, resting) activities. Add the timeframe for each activity. |
| **Measurements** | Daily | Please fill out the time slots in which you have worn the sensors. |
| **Symptoms** | Daily | Please fill out whether you have symptoms that are troublesome in any way during the measurements or activities. If you fall or trip during the measurements, please describe in detail how, where and when this happened. |
| **Not wearing** | If needed | Please document when you did not wear the sensors due to specific reasons (e.g. skin irritation or sensor broken) |
| **Comments** | If needed | Please report here general comments and anything you want to tell us, which was not covered by the domains listed above. |

# Regular medical therapy

| **Medication** | **Dose** | **Unit** | **Frequency** |
| --- | --- | --- | --- |
|  |  | □ g  □ mg  □microg/µg  □ ml  □ Other: |  |
|  |  | □ g  □ mg  □microg/µg  □ ml  □ Other: |  |
|  |  | □ g  □ mg  □microg/µg  □ ml  □ Other: |  |
|  |  | □ g  □ mg  □microg/µg  □ ml  □ Other: |  |
|  |  | □ g  □ mg  □microg/µg  □ ml  □ Other: |  |
|  |  | □ g  □ mg  □microg/µg  □ ml  □ Other: |  |

# Single medication deviations as required

| **Date (DD/MM/YYYY)** | **Medication** | **Dose** | **Unit** |
| --- | --- | --- | --- |
|  |  |  | □ g  □ mg  □ microg/µg  □ ml  □ Other: |
|  |  |  | □ g  □ mg  □ microg/µg  □ ml  □ Other: |
|  |  |  | □ g  □ mg  □ microg/µg  □ ml  □ Other: |
|  |  |  | □ g  □ mg  □ microg/µg  □ ml  □ Other: |
|  |  |  | □ g  □ mg  □ microg/µg  □ ml  □ Other: |

# General guidelines

- The sensors are not waterproof; please take them off when showering. Sweating is no problem for the sensors. Therefore, we ask you to wear the sensors during exhausting daily activities / sports.
- Please document if you have issues with the sensors, for example problems with charging, data transfer or wearing the sensors, and contact your contact person at the local site. Contact details are provided in the user manual. Additionally, you can contact Corina Maetzler in Kiel, Germany. Phone number: +49 431 500 23895, email: corina.maetzler@uksh.de.
- As already stated in the informed consent sheet, all data you measure by wearing the sensors and all written information will be pseudonymized. This means that the movement data can only be related to your personal data in association with a code which will be stored at a safe place that is only accessible to staff of this study. Hence, please do not mention your name in this diary.

Once again, thank you very much for your participation!

# How to complete the activities table

| **Element** | **What to fill out** |
| --- | --- |
| **Activities** | Choose your performed activity and if not listed, please select other and specify your activity. |
| **Description** | Indicate what specific type of activity you perform. Also, document falls, trips or slips, please describe where, when and how it happened |
| **Level of intensity** | Please specify the level of intensity of the performed activity if possible  □ Mild: Being able to speak full sentences at ease  □ Moderate: Being able to speak short sentences  □ Vigorous: Not being able to speak during the activity |
| **Time slot** | Please, roughly indicate the timeslot of activity |
| **Symptoms during activity** | Please indicate if you experienced any PD symptoms during the performance of the activity. |

# Example

| **Activity** | **Description** | **Level of intensity** | **Time slot** | **Symptoms during activity** |
| --- | --- | --- | --- | --- |
| **ACTIVITIES INSIDE THE HOUSE** | | | | |
| ■ **Housekeeping**  □ **Cooking**  □ **Doing crafts**  □ **Working on computer**  □ **Watch TV**  □ **Other:** | *I vacuum cleaned my apartment and I cleaned bathroom.* | ■ Mild  □ Moderate  □ Vigorous | From: *9.00am*  To: *11.00am* | □ Tremor  □ Freezing  ■ Hypokinesia/ slowness  ■ Stiffness  □ Pain  □ Other: |
| **Comment:** *I felt a bit of stiffness during my housekeeping, predominantly on my right arm. I think I was slower than I was last week on the same task, maybe because I did not sleep well last night.* | | | | |

# Activities Day 1

| **Activity** | **Description** | **Level of intensity** | **Time slot** | **Symptoms during activity** |
| --- | --- | --- | --- | --- |
| **ACTIVITIES INSIDE THE HOUSE** | | | | |
| □ **Housekeeping**  □ **Cooking**  □ **Doing crafts**  □ **Working on computer**  □ **Watch TV**  □ **Other:** |  | □ Mild  □ Moderate  □ Vigorous | From:  To: | □ Tremor  □ Freezing  □ Hypokinesia/ slowness  □ Stiffness  □ Pain  □ Other: |
| **Comment:** | | | | |
| □ **Housekeeping**  □ **Cooking**  □ **Doing crafts**  □ **Working on computer**  □ **Watch TV**  □ **Other:** |  | □ Mild  □ Moderate  □ Vigorous | From:  To: | □ Tremor  □ Freezing  □ Hypokinesia/ slowness  □ Stiffness  □ Pain  □ Other: |
| **Comment:** | | | | |
| □ **Housekeeping**  □ **Cooking**  □ **Doing crafts**  □ **Working on computer**  □ **Watch TV**  □ **Other:** |  | □ Mild  □ Moderate  □ Vigorous | From:  To: | □ Tremor  □ Freezing  □ Hypokinesia/ slowness  □ Stiffness  □ Pain  □ Other: |
| **Comment:** | | | | |
| □ **Housekeeping**  □ **Cooking**  □ **Doing crafts**  □ **Working on computer**  □ **Watch TV**  □ **Other:** |  | □ Mild  □ Moderate  □ Vigorous | From:  To: | □ Tremor  □ Freezing  □ Hypokinesia/ slowness  □ Stiffness  □ Pain  □ Other: |
| **Comment:** | | | | |
| □ **Housekeeping**  □ **Cooking**  □ **Doing crafts**  □ **Working on computer**  □ **Watch TV**  □ **Other:** |  | □ Mild  □ Moderate  □ Vigorous | From:  To: | □ Tremor  □ Freezing  □ Hypokinesia/ slowness  □ Stiffness  □ Pain  □ Other: |
| **Comment:** | | | | |
| □ **Housekeeping**  □ **Cooking**  □ **Doing crafts**  □ **Working on computer**  □ **Watch TV**  □ **Other:** |  | □ Mild  □ Moderate  □ Vigorous | From:  To: | □ Tremor  □ Freezing  □ Hypokinesia/ slowness  □ Stiffness  □ Pain  □ Other: |
| **Comment:** | | | | |
| □ **Housekeeping**  □ **Cooking**  □ **Doing crafts**  □ **Working on computer**  □ **Watch TV**  □ **Other:** |  | □ Mild  □ Moderate  □ Vigorous | From:  To: | □ Tremor  □ Freezing  □ Hypokinesia/ slowness  □ Stiffness  □ Pain  □ Other: |
| **Comment:** | | | | |
| □ **Housekeeping**  □ **Cooking**  □ **Doing crafts**  □ **Working on computer**  □ **Watch TV**  □ **Other:** |  | □ Mild  □ Moderate  □ Vigorous | From:  To: | □ Tremor  □ Freezing  □ Hypokinesia/ slowness  □ Stiffness  □ Pain  □ Other: |
| **Comment:** | | | | |

| **Activity** | **Description** | **Level of intensity** | **Time slot** | **Symptoms during activity** |
| --- | --- | --- | --- | --- |
| **ACTIVITES AROUND THE HOUSE** | | | | |
| □ **Gardening**  □ **Tinkering**  □ **Doing crafts**  □ **Other:** |  | □ Mild  □ Moderate  □ Vigorous | From:  To: | □ Tremor  □ Freezing  □ Hypokinesia/ slowness  □ Stiffness  □ Pain  □ Other: |
| **Comment:** | | | | |
| □ **Gardening**  □ **Tinkering**  □ **Doing crafts**  □ **Other:** |  | □ Mild  □ Moderate  □ Vigorous | From:  To: | □ Tremor  □ Freezing  □ Hypokinesia/ slowness  □ Stiffness  □ Pain  □ Other: |
| **Comment:** | | | | |
| □ **Gardening**  □ **Tinkering**  □ **Doing crafts**  □ **Other:** |  | □ Mild  □ Moderate  □ Vigorous | From:  To: | □ Tremor  □ Freezing  □ Hypokinesia/ slowness  □ Stiffness  □ Pain  □ Other: |
| **Comment:** | | | | |
| □ **Gardening**  □ **Tinkering**  □ **Doing crafts**  □ **Other:** |  | □ Mild  □ Moderate  □ Vigorous | From:  To: | □ Tremor  □ Freezing  □ Hypokinesia/ slowness  □ Stiffness  □ Pain  □ Other: |
| **Comment:** | | | | |

| **Activity** | **Description** | **Level of intensity** | **Time slot** | **Symptoms during activity** |
| --- | --- | --- | --- | --- |
| **ACTIVITIES OTHER ENVIRONMENT** | | | | |
| □ **Shopping**  □ **Going for a walk**  □ **Playing sports**  □ **Visiting friends/ family**  □ **Riding a bike**  □ **Other:** |  | □ Mild  □ Moderate  □ Vigorous | From:  To: | □ Tremor  □ Freezing  □ Hypokinesia/ slowness  □ Stiffness  □ Pain  □ Other: |
| **Comment:** | | | | |
| □ **Shopping**  □ **Going for a walk**  □ **Playing sports**  □ **Visiting friends/ family**  □ **Riding a bike**  □ **Other:** |  | □ Mild  □ Moderate  □ Vigorous | From:  To: | □ Tremor  □ Freezing  □ Hypokinesia/ slowness  □ Stiffness  □ Pain  □ Other: |
| **Comment:** | | | | |
| □ **Shopping**  □ **Going for a walk**  □ **Playing sports**  □ **Visiting friends/ family**  □ **Riding a bike**  □ **Other:** |  | □ Mild  □ Moderate  □ Vigorous | From:  To: | □ Tremor  □ Freezing  □ Hypokinesia/ slowness  □ Stiffness  □ Pain  □ Other: |
| **Comment:** | | | | |
| □ **Shopping**  □ **Going for a walk**  □ **Playing sports**  □ **Visiting friends/ family**  □ **Riding a bike**  □ **Other:** |  | □ Mild  □ Moderate  □ Vigorous | From:  To: | □ Tremor  □ Freezing  □ Hypokinesia/ slowness  □ Stiffness  □ Pain  □ Other: |
| **Comment:** | | | | |

| **Activity** | **Description** | **Level of intensity** | | **Time slot** | **Symptoms during activity** |
| --- | --- | --- | --- | --- | --- |
| **USED PUBLIC TRANSPORTATION** | | | | | |
| □ **Car; driver**  □ **Car; passenger**  □ **Bus**  □ **Motor cycle/ scooter**  □ **Train**  □ **Other:** |  | □ Mild  □ Moderate  □ Vigorous | From:  To: | | □ Tremor  □ Freezing  □ Hypokinesia/ slowness  □ Stiffness  □ Pain  □ Other: |
| **Comment:** | | | | | |
| □ **Car; driver**  □ **Car; passenger**  □ **Bus**  □ **Motor cycle/ scooter**  □ **Train**  □ **Other:** |  | □ Mild  □ Moderate  □ Vigorous | From:  To: | | □ Tremor  □ Freezing  □ Hypokinesia/ slowness  □ Stiffness  □ Pain  □ Other: |
| **Comment:** | | | | | |
| □ **Car; driver**  □ **Car; passenger**  □ **Bus**  □ **Motor cycle/ scooter**  □ **Train**  □ **Other:** |  | □ Mild  □ Moderate  □ Vigorous | From:  To: | | □ Tremor  □ Freezing  □ Hypokinesia/ slowness  □ Stiffness  □ Pain  □ Other: |
| **Comment:** | | | | | |
| □ **Car; driver**  □ **Car; passenger**  □ **Bus**  □ **Motor cycle/ scooter**  □ **Train**  □ **Other:** |  | □ Mild  □ Moderate  □ Vigorous | From:  To: | | □ Tremor  □ Freezing  □ Hypokinesia/ slowness  □ Stiffness  □ Pain  □ Other: |
| **Comment:** | | | | | |

| **Activity** | **Description** | **Time slot** | **Symptoms during activity** |
| --- | --- | --- | --- |
| **RESTING ACTIVITIES** | | | |
| □ **Sleeping during the day**  □ **Resting**  □ **Sleeping during the night**  □ **Other:** |  | From:  To: | □ Tremor  □ Freezing  □ Hypokinesia/ slowness  □ Stiffness  □ Pain  □ Other: |
| **Comment:** | | | |
| □ **Sleeping during the day**  □ **Resting**  □ **Sleeping during the night**  □ **Other:** |  | From:  To: | □ Tremor  □ Freezing  □ Hypokinesia/ slowness  □ Stiffness  □ Pain  □ Other: |
| **Comment:** | | | |
| □ **Sleeping during the day**  □ **Resting**  □ **Sleeping during the night**  □ **Other:** |  | From:  To: | □ Tremor  □ Freezing  □ Hypokinesia/ slowness  □ Stiffness  □ Pain  □ Other: |
| **Comment:** | | | |
| □ **Sleeping during the day**  □ **Resting**  □ **Sleeping during the night**  □ **Other:** |  | From:  To: | □ Tremor  □ Freezing  □ Hypokinesia/ slowness  □ Stiffness  □ Pain  □ Other: |
| **Comment:** | | | |

| **EATING** | | | | | |
| --- | --- | --- | --- | --- | --- |
| **Breakfast**  From:  To: | | **Lunch**  From:  To: | | **Dinner**  From:  To: | |
| **SNACKS** | | | | | |
| From:  To: | From:  To: | From:  To: | From:  To: | From:  To: | From:  To: |

| **TIME SLOTS IN WHICH YOU HAVE WORN THE SENSORS** | | | | | |
| --- | --- | --- | --- | --- | --- |
| From:  To: | From:  To: | From:  To: | From:  To: | From:  To: | From:  To: |

# Adverse events from wearing the sensors

| **SENSOR NOT WORN** | |
| --- | --- |
| **Reason** | **Time frame** |
|  | From:  To: |
|  | From:  To: |
|  | From:  To: |
|  | From:  To: |
|  | From:  To: |
|  | From:  To: |
| **GENERAL COMMENTS** | |
| *E.g. Indicate here if you did fall, trip or slip (describe where, when and how it happened)* | |

**Supplementary Figure 1. Physical activity detection separated by tremor presentation**

**
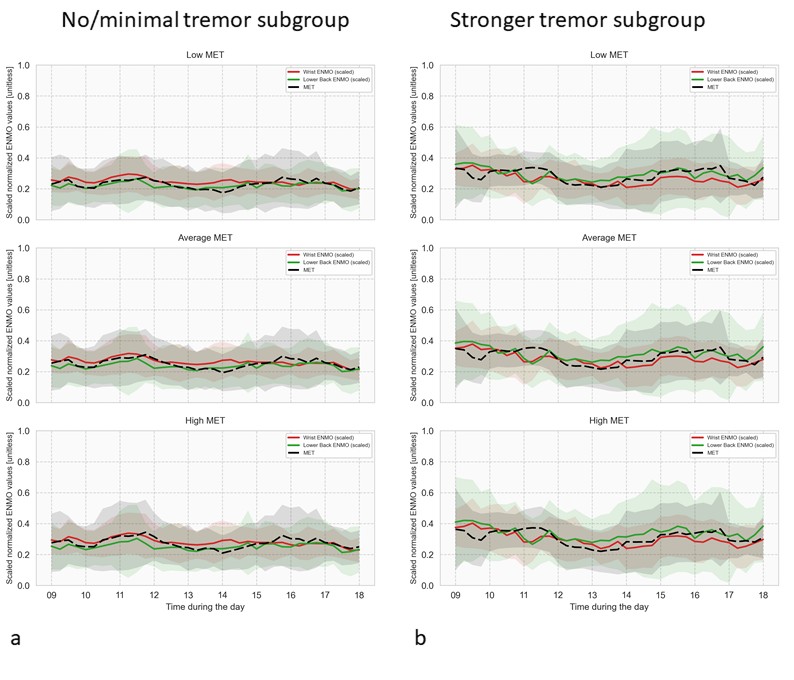
**

***Supplementary Figure 1.*** *Scaled normalized wrist (blue) and lower back (green) inertial measurement units (IMU); Euclidean Norm Minus One (ENMO) values, overlaid with normalized low, average and high MET values (black dashed line), for the subgroup with no/minimal tremor (a, N=14) and the subgroup with stronger tremor (b, N=11). Shaded areas represent ± 1 standard deviation.*
